# Scene consistency enhances state representations of real-world objects

**DOI:** 10.1038/s41598-025-01662-3

**Published:** 2025-05-27

**Authors:** Yuri A. Markov, Melissa Lê-Hoa Võ

**Affiliations:** 1 Department of Psychology, Goethe University Frankfurt, Frankfurt am Main, Germany; 2 Department of Psychology, Ludwig-Maximilians-Universität München, Munich, Germany

**Keywords:** Object recognition, Scene context, Consistency effect, Real-world objects, Objects states, Human behaviour, Attention, Perception

## Abstract

Previous research has shown that the context in which objects are located significantly influences how efficiently they are categorized. However, less is known about whether scene consistency can also affect the processing of finer object features, such as the state of an object (e.g., the angle of a Swiss army knife or the fill level of a bottle). Therefore, across three experiments, we presented a subset of the JURICS stimulus set, in which each object exists in 20 continuously varying states (e.g., from fully closed to fully open) in scenes that were either contextually consistent or inconsistent. Participants were asked to report the specific state of the object using a continuous report task. Our results showed that scene consistency enhanced the precision of state judgments; that is, participants made significantly larger errors in reporting object states when objects were presented in inconsistent compared to consistent scenes. These findings suggest that scene context exhibits its effect already at the level of fine-grained perceptual processing of objects, affecting not only object categorization but also the accuracy of its perceived features.

**Supplementary Information:**

The online version contains supplementary material available at 10.1038/s41598-025-01662-3.

## Introduction

Human vision is limited, yet we are able to see the world continuously and in rich detail. This proficiency is largely due to our ability to use context to fill in perceptual gaps and make sense of what we see. Context not only aids in recognizing objects but also shapes our entire visual experience, drawing on both current surroundings and accumulated knowledge^1–6^.

Early studies focused on the influence of context on object recognition using line drawings and a forced-choice paradigm^7–9^, and demonstrated the facilitation of object recognition in consistent contexts. However, later studies criticized this paradigm, finding it difficult to replicate the effect^10,11^. Nevertheless, subsequent studies using object naming tasks were able to demonstrate similar facilitation—the *scene consistency effect*^4,6,12–16^. They showed that objects in consistent contexts were named more accurately. Moreover, it was shown that not only does consistent context cause facilitation, but also that inconsistent context impedes object processing^12^.

But how does context affect early object processing? Event-related potential studies have suggested that scene consistency violations elicit N300/400 responses^12,17–22^. The N400 ERP component is known to signal semantic processing, while the N300 is associated with more perceptual processing, preceding object identification^21–23^. Later fMRI^24^ and TMS^25^ studies provided further evidence that scene context influences early visual object representations. Visually degraded objects elicited patterns similar to original objects only when they were presented in consistent scenes. Impairment of object recognition, on the other hand, occurred when the occipital place area (OPA) and lateral occipital cortex (LOC) were causally stimulated, suggesting that feedback mechanisms underlie the scene consistency effect, influencing representations in early visual cortex. This is supported by recent behavioral research, which has demonstrated that scene consistency influences the subjective perception of an image’s noisiness^26^.

So far, most studies have primarily aimed to understand how contextual regularities facilitate perceptual stages of scene processing as a whole. For instance, Mudrik^21,23^ and colleagues used congruent and incongruent action scenes (e.g., people playing with a basketball vs. people playing with a watermelon), demonstrating that observers were able to more precisely report the number of hands involved in the action when presented in consistent scene contexts, while also showing modulations of the N300/400 components. It is important to highlight that these studies primarily addressed action-related context rather than object-specific context. However, there has been no direct demonstration that scene consistency can affect the percepts of objects themselves.

**Fig. 1:**
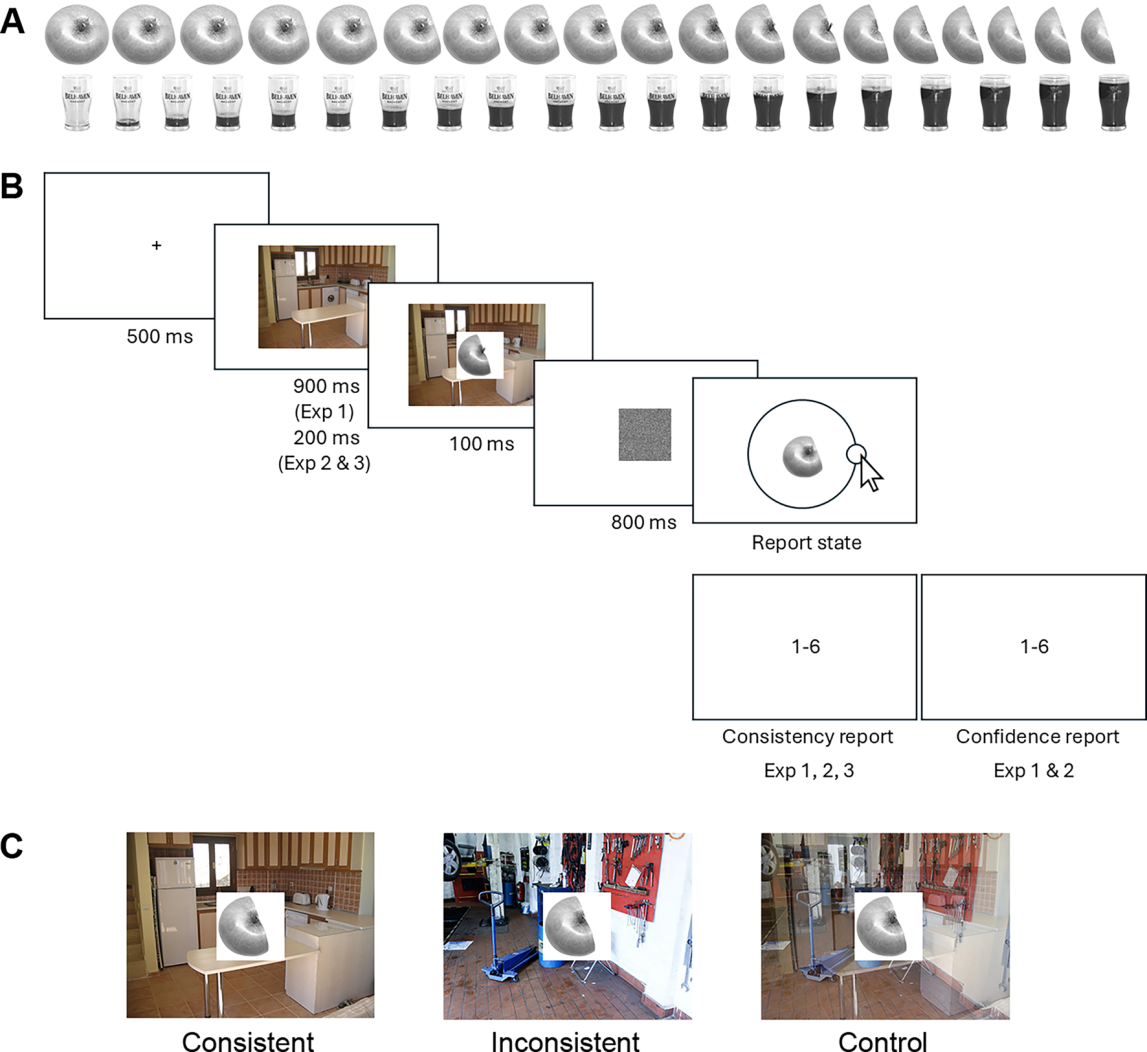
(**A**) Example stimuli from the JURICS stimulus base^27^; objects change state in 20 continuous steps. (**B**) The trial sequence for Experiments 1, 2, and 3. First, a scene alone was presented, followed by the brief presentation of the target object embedded within the scene. Thereafter, a dynamic white noise mask appeared. Participants then adjusted the object’s state using a continuous report task and rated object-scene consistency and confidence. After completing the continuous report task, participants were asked to rate the scene-object consistency and their confidence on a scale from 1 to 6, where 1 indicated low consistency/confidence and 6 indicated high consistency/confidence. (**C**) Three example object-scene combinations (experimental conditions): consistent, inconsistent, and control. The stimuli shown in the figure are not drawn to scale.

Here, we aim to explore how context can affect our ability to process object features efficiently and precisely. We chose the state of the object (e.g., to what degree a laptop is open or a glass is full; see Fig. 1A) as a realistic and ecologically valid feature that varies across instances^27^. The state of an object refers to any specific change in the object, such as a change in condition, position, status, or form, leading to a change in its physical properties, configuration, or appearance. We presented objects in consistent and inconsistent scenes and asked observers to report the continuous state of the object.

## Experiment 1

Participants reported the states of objects placed in both consistent and inconsistent scenes using a continuous report task (Fig. 1B). Objects within a scene were briefly presented, followed by a mask and a continuous report task, in which observers adjusted the state of the object to match the state of the previously presented object. Each object was repeated twice during the experiment under two different conditions. After the state report, observers also reported scene-object consistency and their confidence in the report. We expected to find smaller adjustment errors for objects presented in consistent scenes. The experiment was preregistered at https://osf.io/ejzd6.

### Results and discussion

#### Mean error

We analyzed the mean error of adjustment as the difference in “frames” between the reported state and the originally presented state. Since the error distribution consisted of integer values ranging from 0 to 19 and was right-skewed, we used a negative binomial generalized linear mixed model (GLMM) for analysis. The dependent variable was the error, the independent variable was the condition, and random intercepts were included for participant, object name, and presented state (see the Analysis section for more details).

**Fig. 2:**
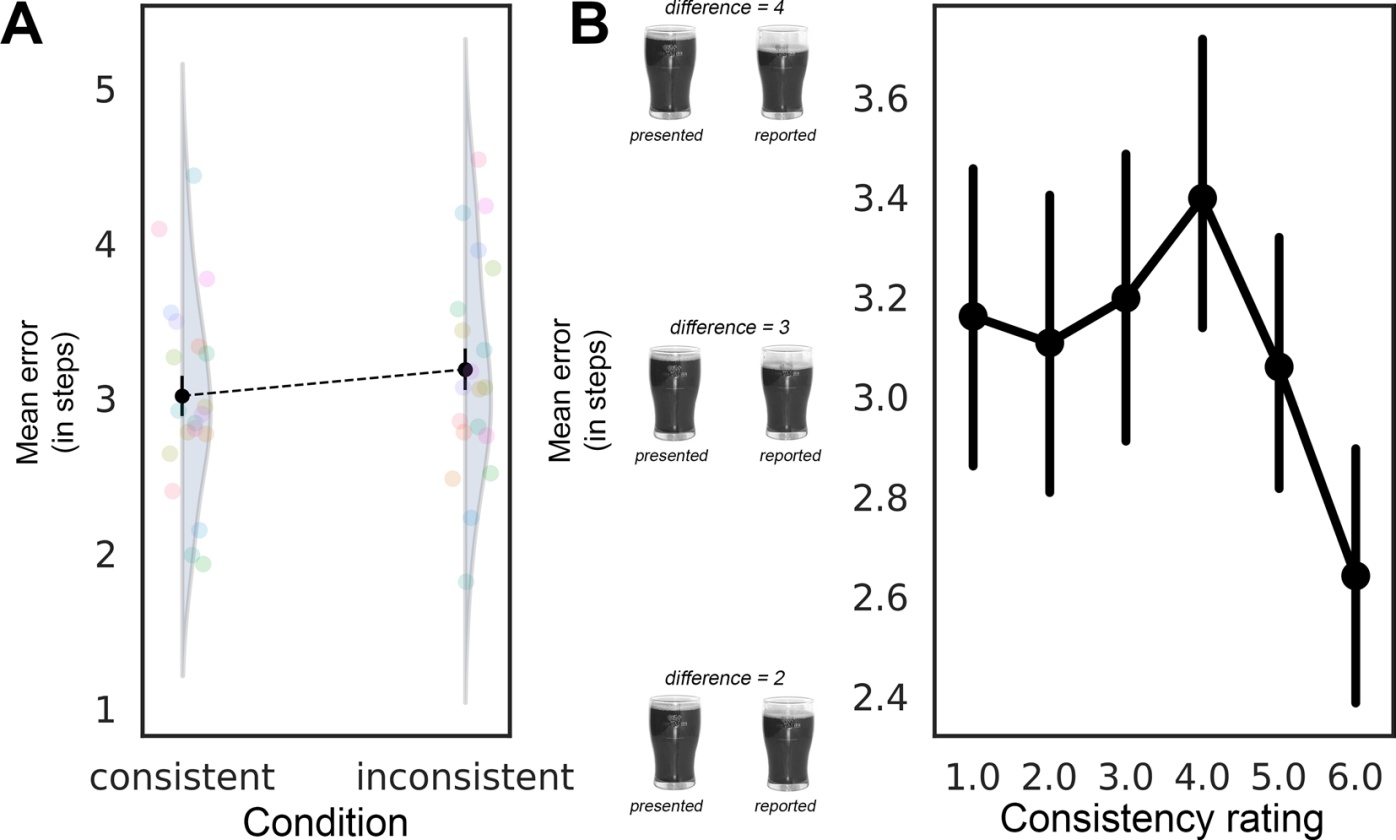
(**A**) Mean error as a function of conditions; (**B**) Mean error as consistency reports ratings. Transparent colored circles represent individual participants’ data points. Error bars depict 95% CIs.

The effect of condition was not statistically significant (*β* = 0.054, *SE* = 0.033, *z* = 1.617, *p* = 0.106; see Fig. 2A), suggesting that the experimental condition did not substantially influence error rates (although there was a small effect according to an RM-ANOVA: *p* = 0.043, see Supplementary Materials). Additionally, we analyzed the effect of individual observers’ consistency reports on the mean error. The consistency reports predictor was significant (*β* =  − 0.026, *SE* = 0.011, *z* =  − 2.475, *p* = 0.013; see Fig. 2B), suggesting that higher subjective consistency report values were associated with a decrease in errors.

We calculated the proportion of variance explained by the fixed effects for both condition and consistency reports as independent variables^28^. We found that observers’ individual consistency reports had a larger effect size (R^2^_m_ = 0.002) compared to the pre-defined condition (R^2^_m_ = 0.001), suggesting that individual differences in consistency reports were a better predictor of error rates than the experimental condition.

#### Consistency and confidence reports

Participants reported that consistent scenes were more consistent compared to inconsistent ones (consistent: *M* = 4.37, *SD* = 1.45; inconsistent: *M* = 2.82, *SD* = 1.51; comparison: *t*(19) = 6.6, *p* < 0.001, Cohen’s d = 2.13). Confidence reports were slightly higher for consistent compared to inconsistent condition (*t*(19) = 2.8, *p* = 0.011, Cohen’s d = 0.225).

In Experiment 1, we found that scene consistency did not have a strong direct effect on the precision of reported object states. While a small effect was detected in an RM ANOVA (*p* = 0.043, see Supplementary Materials), the negative binomial GLMM did not show a statistically significant effect of condition on error rates, suggesting that scene consistency alone does not substantially influence the accuracy of object state reports. However, observers’ consistency reports were a stronger predictor of error (*p* = 0.013). These results suggest that an observer’s subjective consistency evaluation can affect object perception at the level of state representation.

## Experiment 2

In Experiment 1, we found indications that consistency affected the perception of the object, but were unable to disentangle whether the consistency effect was caused by facilitation or interference^12^. In Experiment 2, we therefore added a baseline condition in which we overlaid scenes from the consistent and inconsistent conditions onto each other. Based on previous studies, we expected the scene consistency effects to be caused both by facilitation and interference. The experiment was preregistered at https://osf.io/duysm.

### Results and discussion

#### Mean error

**Fig. 3:**
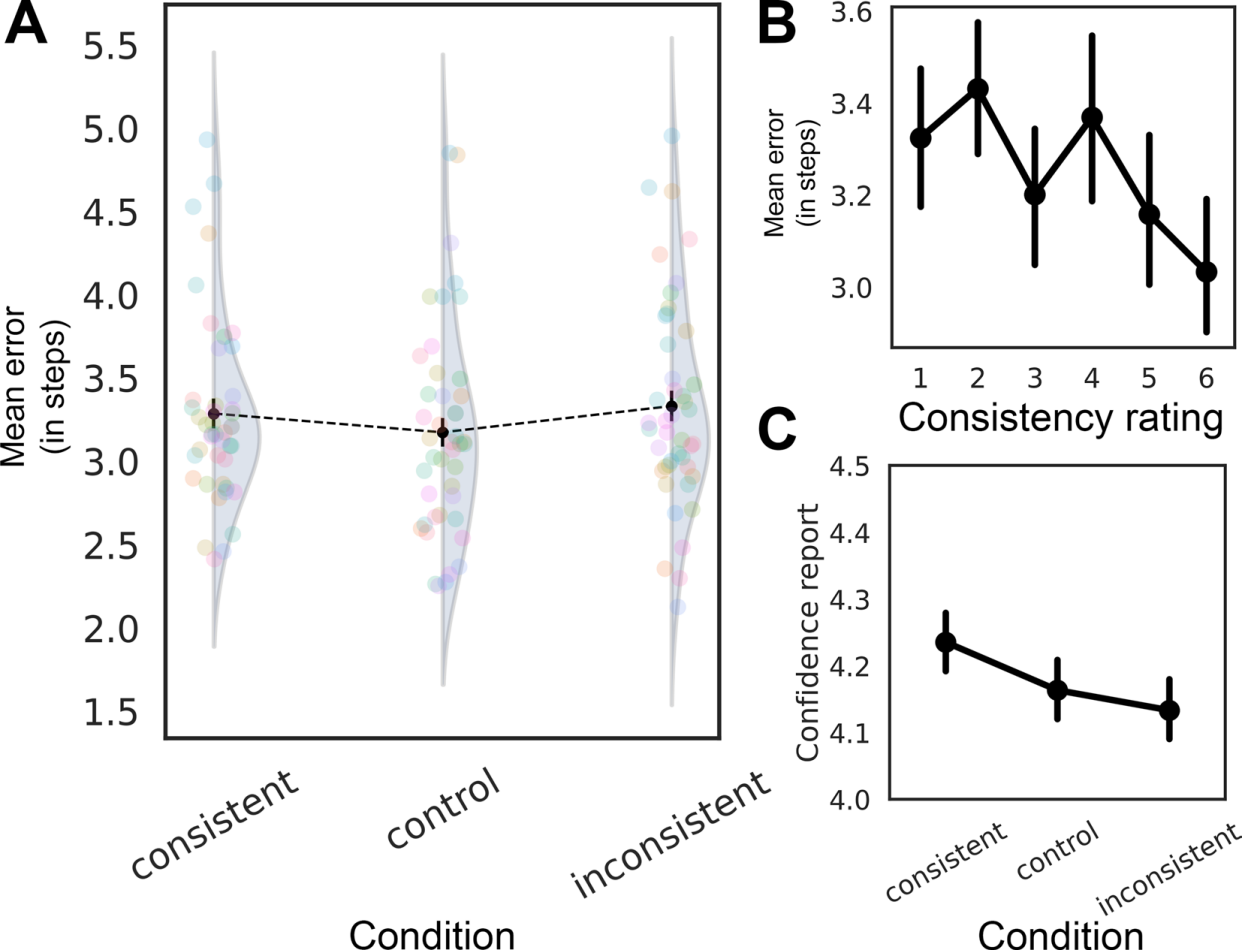
(**A**) Mean error as a function of conditions; (**B**) Mean error as a function of consistency reports ratings; (**C**) Confidence reports as a function of conditions. Error bars depict 95% CIs.

To evaluate whether the experimental condition influenced the precision of state adjustment, we compared a full model including condition as a fixed effect with a null model that included only random intercepts. A likelihood ratio test (LRT) indicated that adding condition did not significantly improve model fit (*χ*^2^(2) = 5.03, *p* = 0.081; see Fig. 3A). This suggests that condition, as a whole, did not have a strong effect on error rates.

We then ran the GLMM with the control condition as the baseline. The consistent condition did not significantly differ from the control (*β* = 0.029, *SE* = 0.022, *z* = 1.345, *p* = 0.179), suggesting that scene consistency did not strongly benefit performance. At the same time, the inconsistent condition led to significantly higher errors compared to the control condition (*β* = 0.049, *SE* = 0.022, *z* = 2.228, *p* = 0.026), indicating that scene inconsistency impaired performance.

As in Experiment 1, we tested a second model including slider consistency reports as an independent variable to explore alternative predictors. The effect of the slider response was statistically significant (*β* =  − 0.020, *SE* = 0.006, *z* =  − 3.57, *p* < 0.001; see Fig. 3B), suggesting that higher consistency ratings were associated with lower error rates. The effect sizes were comparable to those in Experiment 1 – the consistency ratings model had R^2^_m_ = 0.002, and the condition model had R^2^_m_ = 0.001.

#### Consistency and confidence reports

As one would expect, subjective consistency reports were affected by our object-scene consistency manipulation (consistent: *M* = 4.27, *SD* = 1.58; control: *M* = 3.31, *SD* = 1.65; inconsistent: *M* = 2.35, *SD* = 1.38; comparison: RM ANOVA, *F*(2, 86) = 146.6, *p* < 0.001, η^2^_p_ = 0.77) in that participants reported that consistent scenes were more consistent compared to both the inconsistent (*t*(43) = 12.38, *p*_*holm*_ < 0.001) and control conditions (*t*(43) = 12.58, *p*_*holm*_ < 0.001), while inconsistent trials had the lowest consistency ratings (*t*(43) = 10.82, *p*_*holm*_ < 0.001).

Additionally, we found a significant effect of object-scene consistency on confidence reports (Fig. 3C, RM ANOVA, *F*(2, 86) = 5.84, *p* = 0.004, η^2^_p_ = 0.12), with higher reported confidence for consistent trials (consistent vs. inconsistent comparison: *t*(43) = 3.2, *p*_*holm*_ = 0.007).

Thus, we found rather weak evidence that scene consistency affects the precision of observers’ reports, while its effects on confidence were more robust.

**Fig. 4:**
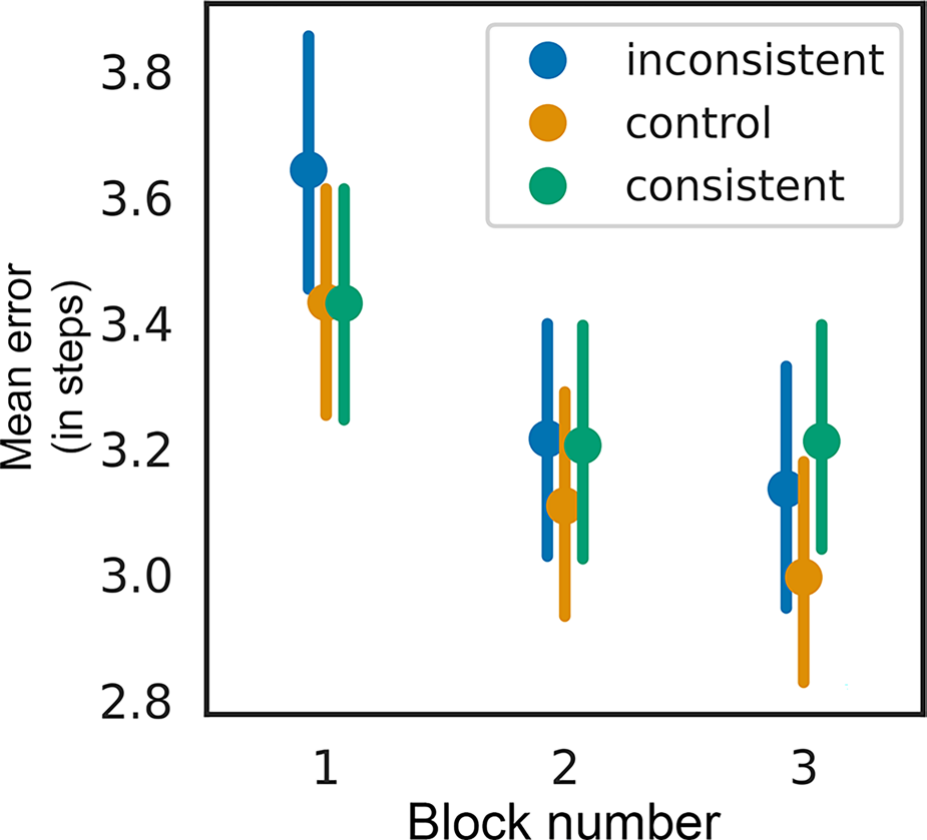
Mean error as a function of the block number and condition. Error bars depict 95% CIs.

Importantly, we observed that subjective consistency reports predicted adjustment errors more accurately than experimental conditions. Nevertheless, the effects were much smaller than expected. To understand the underlying reasons for these weak effects, we conducted an exploratory analysis. We hypothesized that object repetitions could have influenced the precision of observers’ reports, potentially leading to ceiling effects in adjustments or shaping certain expectations for observers (Fig. 4). We ran a GLMM including the block number as the fixed effect. The results revealed a significant effect of block number on error rates (*β* =  − 0.067, *SE* = 0.011, *z* =  − 6.145, *p* < 0.001), indicating that errors decreased across blocks. Repeating the target—even in a different state—appears to have substantially increased familiarity with the object, leading to improved performance^29,30^.

We therefore analyzed data from block 1 alone. A likelihood ratio test (LRT) indicated that adding condition significantly improved model fit (*χ*^2^(2) = 7.19, *p* = 0.027). This suggests that object-scene consistency had a significant effect on error rates during the first block. The consistent condition did not significantly differ from the control condition (*β* =  − 0.007, *SE* = 0.037, *z* =  − 0.192, *p* = 0.848), but the inconsistent condition led to a significant increase in error rates compared to the control condition (*β* = 0.082, *SE* = 0.038, *z* = 2.190, *p* = 0.029), suggesting that scene inconsistency negatively impacted performance during the first block. Models with consistency reports as fixed effects revealed that higher consistency reports were associated with lower error rates (*β* =  − 0.032, SE = 0.010, *z* =  − 3.38, *p* = 0.0007). Compared to the analysis including all blocks, effect sizes in block 1 were higher for both condition (R^2^_m_ = 0.002) and consistency report (R^2^_m_ = 0.004) models (compare to R^2^_m_ = 0.001 and R^2^_m_ = 0.002 in all blocks analysis).

Moreover, the repetition of objects likely also created certain expectations, potentially diminishing the consistency effect. Considering these factors, we conducted a third experiment in which each object was presented only once, to test whether the results of Experiments 1 and 2 would hold—or even increase in strength.

## Experiment 3

Due to the potential ceiling effects of repeatedly presenting the same target, we presented each target only once in Experiment 3. However, this approach meant that different observers encountered different objects under different conditions. To control for differences in adjustment precision across objects, we divided all objects into three groups with similar mean adjustment errors (based on data from Experiments 1 and 2). Each group of objects was then presented under either consistent, control, or inconsistent conditions, counterbalanced across participants.

### Results and discussion

#### Mean error

**Fig. 5:**
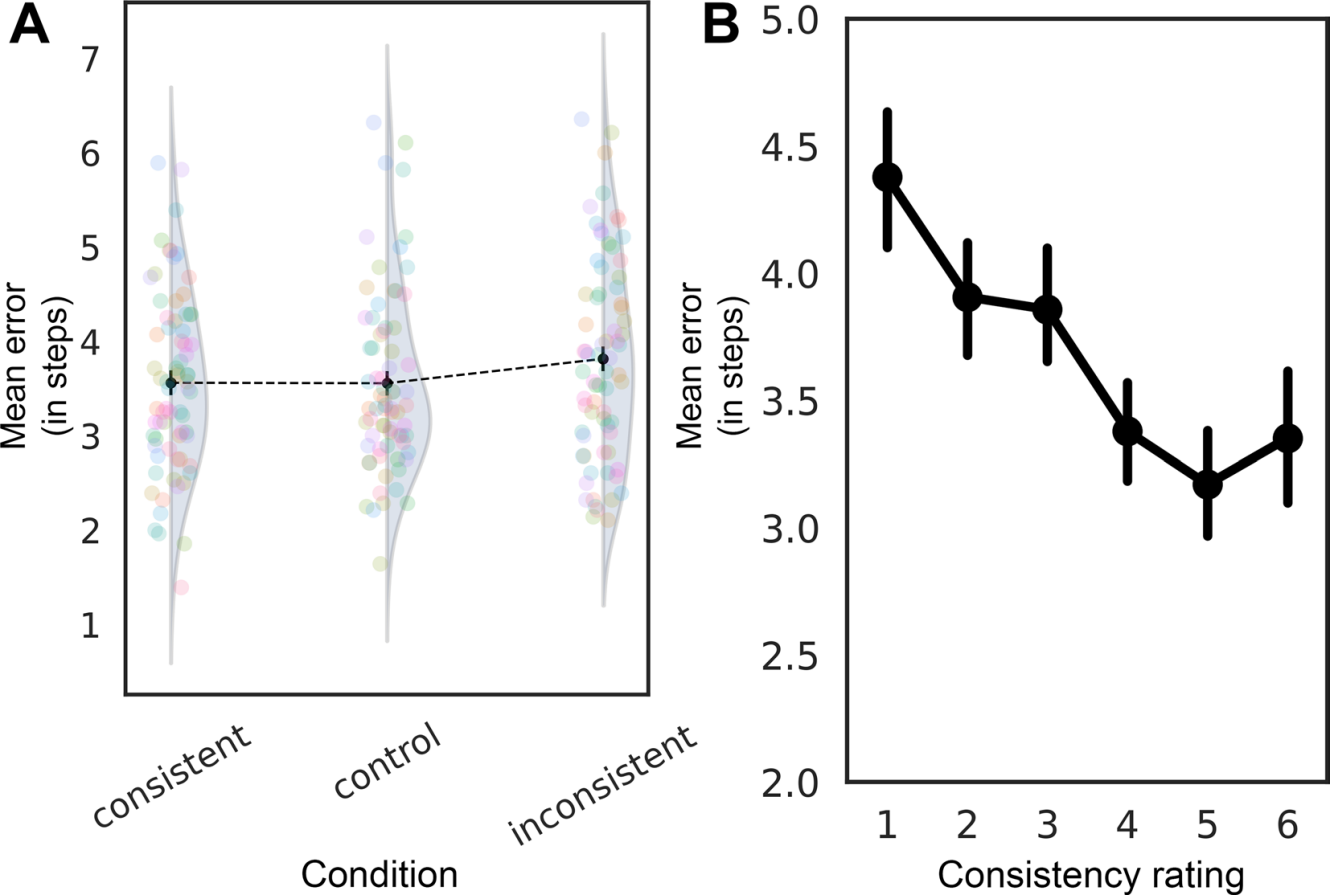
(**A**) Mean error as a function of conditions; (**B**) Mean error as a function of consistency reports ratings. Error bars depict 95% CIs.

We ran a GLMM with the control condition as the baseline. A likelihood ratio test (LRT) indicated that adding the object-scene consistency condition to the null model significantly improved model fit (*χ*^2^(2) = 9.97, *p* = 0.007). This suggests that object-scene consistency had a significant effect on mean error. As in Experiments 1 and 2, the consistent condition did not significantly differ from the control condition (*β* = 0.003, *SE* = 0.029, *z* = 0.099, *p* = 0.921, see Fig. 5A), indicating that a consistent scene did not improve object perception. The inconsistent condition, however, led to a significantly higher mean error (*β* = 0.081, *SE* = 0.029, *z* = 2.773, *p* = 0.006, see Fig. 5A), suggesting that scene inconsistency negatively impacted object state reporting.

Higher slider consistency reports were associated with lower mean error (*β* =  − 0.044, *SE* = 0.008, *z* =  − 5.33, *p* < 0.001, see Fig. 5B), indicating that trials perceived as more consistent produced fewer errors.

Compared to the previous experiments (R^2^_m_ = 0.001), effect sizes were higher for both the condition model (R^2^_m_ = 0.002) and the consistency report model (R^2^_m_ = 0.006).

#### Consistency reports

Again, as expected, consistency reports were affected by consistency condition (consistent: *M* = 4.09, *SD* = 1.48; control: *M* = 3.5, *SD* = 1.49; inconsistent: *M* = 2.91, *SD* = 1.48; comparison: RM ANOVA, *F*(2, 142) = 57.98, *p* < 0.001, η^2^_p_ = 0.45), such that consistent scenes were reported as more consistent compared to both the inconsistent (*t*(71) = 8.15, *p*_*holm*_ < 0.001) and control conditions (*t*(71) = 7.17, *p*_*holm*_ < 0.001), while inconsistent trials produced the lowest consistency ratings (*t*(71) = 6.47, *p*_*holm*_ < 0.001).

### Experiments 2 and 3 combined analysis

To increase the power of our analysis, we combined data from Experiments 2 and 3, including only the first block of Experiment 2 and excluding categories not presented in Experiment 3 (“glasses,” “medicine,” and “box”). This resulted in a sample size of 116 participants.

**Fig. 6:**
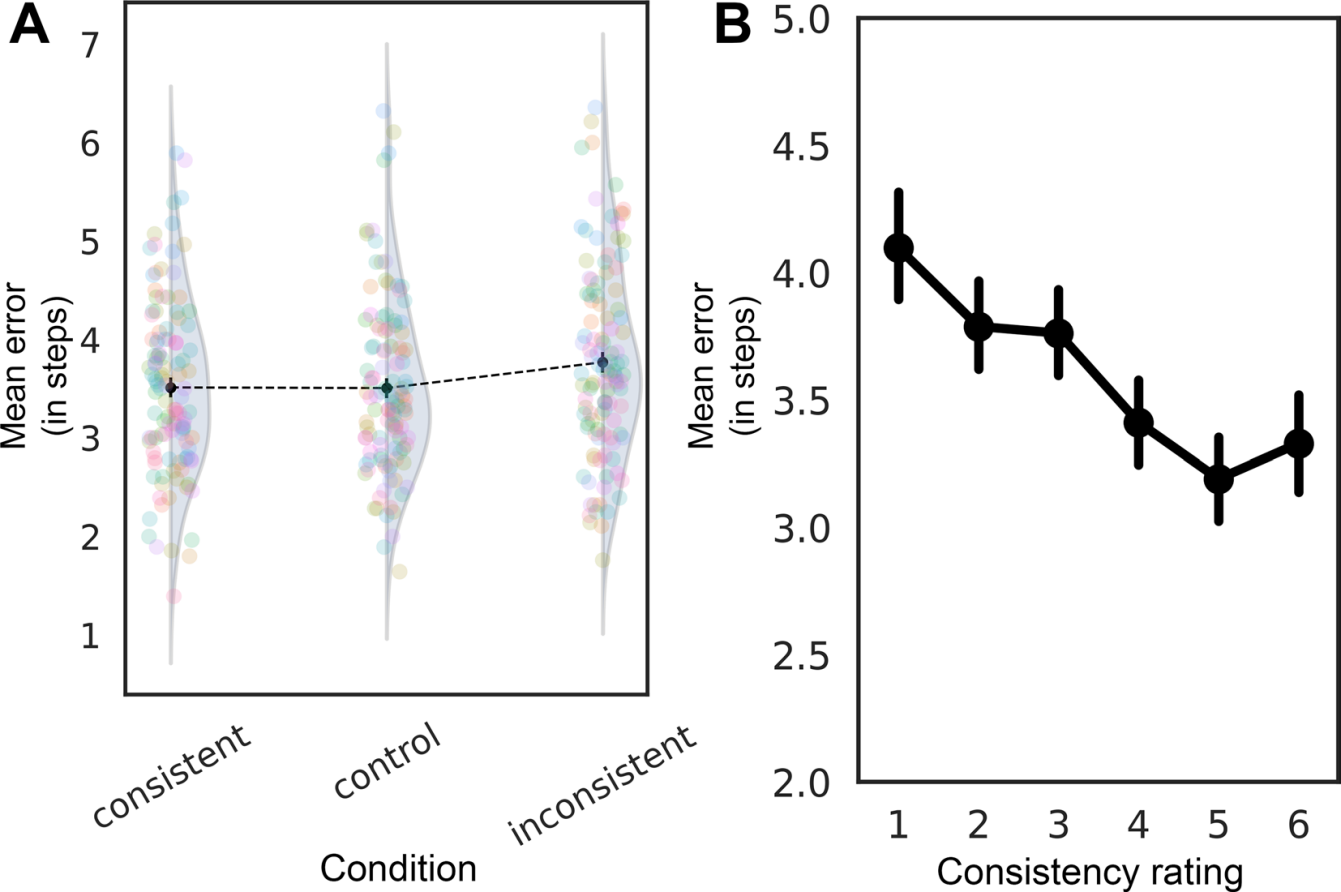
Combined data from Experiment 2 and 3: (**A**) (left) Mean error as a function of conditions; (**B**) Mean error as consistency reports ratings. Error bars depict 95% CIs.

We compared a full GLMM that included condition as a fixed effect to a null model with only random intercepts. A likelihood ratio test (LRT) revealed that adding condition significantly improved model fit (*χ*^2^(2) = 19.30, *p* < 0.001), indicating that condition had a significant effect on mean error. The consistent condition did not significantly differ from the control condition (*β* =  − 0.002, *SE* = 0.023, *z* =  − 0.076, *p* = 0.940, see Fig. 6A). The inconsistent condition, however, led to a significantly higher mean error (*β* = 0.086, *SE* = 0.023, *z* = 3.743, *p* < 0.001, see Fig. 6A), indicating that scene inconsistency negatively affected object state perception. Higher consistency ratings were associated with lower mean error (*β* =  − 0.040, *SE* = 0.006, *z* =  − 6.37, *p* < 0.001, see Fig. 6B). Effect sizes were the same as in Experiment 3 for both the condition model (R^2^_m_ = 0.002) and the consistency report model (R^2^_m_ = 0.006).

### General discussion

Previous studies have demonstrated that scene consistency affects object categorization and identification globally^4,6,12–16^. The present study extends this work by demonstrating a direct influence of scene context on the precision of object state representations. We found that presenting objects in consistent or inconsistent scenes affected the precision of reports about the objects’ states. We also found that consistency influenced observers’ confidence ratings. Most notably, we observed an impairment caused by inconsistent scenes: adjustment error increased in the inconsistent condition compared to the control and consistent conditions, the latter of which did not differ. However, analyses based on self-reported consistency clearly demonstrated both facilitation and interference effects—errors decreased linearly with increasing perceived consistency between scene and object. Thus, we were not only able to replicate previous findings showing inconsistency effects on semantic integration^12,16^, but also to expand them beyond categorization and demonstrate that scene consistency affects visual perception at the level of fine-grained object features.

Our results indicate that both experimentally predefined scene consistency and subjective object-scene consistency reports significantly influenced error rates. However, the effect sizes were relatively small, suggesting that while these factors play a role, they account for only a limited portion of the variance in performance. Despite the small effect sizes, these findings remain meaningful^31–33^, as even subtle influences can shape perceptual and cognitive processing. Moreover, we replicated these results repeatedly across all three experiments, with object-scene consistency reports consistently emerging as a significant predictor of performance. This suggests that individually learned associations between objects and scenes may play a stronger role in object state perception than experimentally predefined scene consistency.

What process could underlie the influence of context on object state processing? According to the MEG/fMRI study^24^, scene context enhances the neural representation and visual recognition of degraded objects by involving a feedback pathway. In this pathway, scene-selective areas such as the retrosplenial cortex (RSC) and parahippocampal place area (PPA) relay scene information back to object-selective regions in the visual cortex, including the lateral occipital cortex (LOC) and posterior fusiform gyrus (pFs). A later study confirmed this hypothesis^25^, using TMS to demonstrate causal early support from the occipital place area (OPA) to object recognition, followed by the LOC. This interaction between scene and object processing pathways supports the facilitation of object representations by congruent scene information, especially when object information is degraded. The feedback not only decreases perceived object noisiness^26^ but also, as in our results, sharpens the object representation itself.

Previous studies have demonstrated the influence of scene consistency on global object identification and categorization^4,6,12–16^. Our results go beyond this work by demonstrating a direct influence of scene context on the precision of object state representations—a finding that is supported by earlier studies showing context effects at a more holistic level^21,23^.

It seems that subjective reports of object-scene consistency were better predictors of error than experimenter-defined conditions. This may be because the experimenters’ own expectations and prior experiences regarding object-scene consistency might not mirror those of all participants—a common issue in such studies. Individual consistency ratings are likely better predictors because they reflect personal experience and prior knowledge about the contexts in which objects typically appear. Thus, they are more likely to capture the subjective influence of context on object processing than predefined experimental conditions. Additionally, participants come from diverse cultural backgrounds, which have been shown to affect scene processing as well^34–37^.

This is not only the first demonstration that context affects the detailed perceptual processing of object representations, but also the first study to use a continuous report state paradigm to investigate the effects of object-scene interactions. The JURICS stimulus set^27^ was designed specifically for continuous report tasks aimed at investigating real-world object representations. Previous studies have already used the state feature as a measure to understand object representations per se^38–42^. Other continuous report stimulus sets—such as Scene Wheels^43^ and Loop Route^44^—also exist, but they focus on scene rather than object stimuli. Thus, the JURICS stimulus set enables the use of more realistic stimuli in continuous report paradigms targeting object-level processing.

### Limitations

While the present study provides novel and important insights into how scene context influences the precision of object state perception, several methodological and interpretive limitations should be acknowledged. These limitations may help explain the relatively small effect sizes observed and point to directions for refining experimental designs in future research. One possible reason for the small effect sizes is the state report task itself, which involved 20 response alternatives. While involving 20 object states, this response format may still be too limited and may not provide the most precise measure for capturing fine-grained influences of scene consistency.

Further, Experiment 2 revealed that repeated presentations of an object reduced the effect of scene-object consistency. First, repetition improved performance in the state report task, likely due to increased familiarity with both the object and the task itself^29,30^. Second, repeated presentation of the same object within the experiment may have increased interference in long-term memory^42^, leading to confusion errors and potentially diminishing the influence of scene-object consistency.

Moreover, in addition to reporting the object’s state, observers were required to process and report the consistency between the object and the scene. This introduces a specific task context, which could potentially influence object processing^45^, as participants needed to distribute their attention across both the object and the scene. The presence of this consistency judgment requirement may have influenced how participants encoded or maintained information. While our study provides evidence for a relationship between object state and scene consistency, the precise role of task context in shaping these effects remains an open question that requires further investigation.

Our experimental conditions showed that scene inconsistency reliably increased mean error—an effect evident when comparing inconsistent trials to a control condition. In contrast, facilitation effects were not as clearly observed at the condition level. While we observed a clear linear relationship between subjective consistency ratings and adjustment error, we acknowledge that interpreting this relationship in terms of facilitation and interference requires caution. The absence of a categorical boundary makes it difficult to pinpoint where interference ends and facilitation begins. We, therefore, interpret the influence of consistency as graded and continuous, shaped by both the experimental context and participants’ subjective experience.

## Conclusion

In conclusion, our findings provide compelling evidence that scene context influences object processing at perceptually detailed levels—enhancing object representations when the context is consistent, and impairing them when it is inconsistent. Methodologically, this study offers new avenues for exploring the complex relationship between scene context and fine-grained object perception using more ecologically valid stimuli.

## Methods

### Participants

Twenty-one participants (20 after exclusion; 6 female; mean age = 28.3 years, SD = 5.9) in Experiment 1, forty-five participants (44 after exclusion; 22 female; mean age = 27.54 years, SD = 5.7) in Experiment 2, and seventy-five participants (72 after exclusion; 23 female; mean age = 29.86 years, SD = 5.5) in Experiment 3 were recruited through the Prolific platform (www.prolific.ac;^46,47^) and given access to the online experiments via Pavlovia (https://pavlovia.org). Participants reported normal or corrected-to-normal vision. Compensation for participation amounted to £9 per hour (Experiment 1 lasted approximately 30 min; Experiment 2, approximately 45 min; Experiment 3, approximately 20 min). Participants gave informed consent as part of Prolific’s standard recruitment process, which includes a pre-screening consent form to ensure understanding and voluntary participation. The experimental procedure conformed to the Declaration of Helsinki and was approved by the local ethics committee of the Faculty of Psychology and Sports Sciences (2014-106R1) at Goethe University Frankfurt.

The sample size for Experiment 1 was based on previous studies with similar continuous report paradigms, however, this is the first study to employ continuous state reports^43,48–52^. In Experiment 1, we excluded data from one participant (resulting in a final sample of 20 participants) because their mean error exceeded 6.6. This threshold was estimated by simulating random responses on each trial; thus, participants with error values above this cutoff were considered either imprecise in task performance or affected by technical difficulties.

For Experiment 2, a power analysis based on the results of Experiment 1 indicated that, for an effect size of Cohen’s *d* = 0.48, at least 44 participants were needed for a RM ANOVA design with three conditions^53^. Importantly, although we had preregistered the use of RM ANOVA, we decided to switch to a GLMM approach due to the continuous scale of the error rates. For completeness, we included the RM ANOVA results in the Supplementary Materials. As in Experiment 1, we excluded data from one participant due to low performance, resulting in a final sample of 44 participants.

For Experiment 3, power analysis based on Experiment 2 showed that for the effect size of η^2^ = 0.068, we need at least 68 participants for the RM ANOVA design with three conditions^53^. To account for potential technical issues or low performance, we decided to collect data from 75 participants. We excluded data from three participants due to low performance, resulting in a final sample of 72 participants—with 24 participants per group/condition.

### Apparatus and stimuli

The experiment was developed and presented online via PsychoPy v2023.1.3^54,55^.

Object stimuli were taken from the JURICS stimulus base^27^, with 87 objects selected from a total of 98, based on cultural relevance (e.g., removing unfamiliar categories like “glazing curd”) and the exclusion of sensitive images (e.g., “cigarettes” and “syringes”). Each object had 20 different states (e.g., a glass filling with juice or a laptop opening). To remove color cues, all object images were converted to grayscale (Fig. 1A).

Scene images were taken from Lauer et al.^56^, the BOiS stimuli set^57^, and Creative Commons-licensed images found through Google search. Scenes were categorized into seven groups: bathroom, children’s room, kitchen, garage, hall, office, and medical cabinet. Each image had a resolution of 1024 × 768 pixels. We manually matched scenes to objects for consistent trials (e.g., a bell pepper in a kitchen). Inconsistent trials were created by using the same set of scenes but pairing them with semantically incongruent objects (e.g., a bell pepper in a bathroom). For the control condition used in Experiments 2 and 3, we overlaid consistent and inconsistent scene images with 50% opacity (e.g., a bell pepper over a blended kitchen/bathroom background). The complete set of 87 scene stimuli has been made available as open-access material on OSF: https://osf.io/kb8my/.

We analyzed both the global frequency structure and local pixel composition of scene images to assess low-level visual similarity. Using Fast Fourier Transform (FFT) magnitude spectra, we compared each object’s corresponding consistent, control, and inconsistent scenes across three conditions: (1) Consistent vs. Control, (2) Consistent vs. Inconsistent, and (3) Inconsistent vs. Control. We computed Pearson correlation, Mean Squared Error (MSE), and Structural Similarity Index (SSIM) to quantify differences in frequency content. Additionally, we conducted pixel-wise correlation to assess direct intensity similarities (see Table 1).

**Table 1:** Image comparison in fast fourier transform mean pearson correlation, mean squared error, structural similarity index, and pixel mean correlation.

Comparison	FFT mean correlation	FFT mean MSE	FFT mean SSIM	Pixel mean correlation
Consistent versus control	0.735	1.026	0.387	0.734
Consistent versus Inconsistent	0.639	1.435	0.25	0.102
Inconsistent versus control	0.735	0.997	0.388	0.728

We found high mean correlations (0.735 and 0.735) for both Consistent vs. Control and Inconsistent vs. Control comparisons, suggesting that the control images retained the spatial frequency characteristics of the original scenes. The MSE values (~ 1.0) indicate a moderate level of pixel-wise differences, while SSIM values (~ 0.39) suggest that, perceptually, the control images diverged from the originals. We found lower similarity in all values between Consistent vs Inconsistent image. This was, however, expected, since the control images were created by blending the consistent and inconsistent images with equal weighting. The pixel-wise correlation between the control and the original images is also high (0.734 for consistent, 0.728 for inconsistent), indicating that the control images maintained an equal blend of pixel values from both conditions.

It is important to note that the stimuli were presented in the middle of the screen and on a white background on top of the scene. Therefore, scene image parameters should not influence object-background segmentation.

We used a virtual chin-rest procedure^58^, in which observers adjusted a bank card shown on the screen to match its physical size. This allowed us to present stimuli in approximately equal visual degrees across different devices. The size of the scene image was 24° × 18°, and the object’s size was 8°. The size of the mask matched the size of the object image. The diameter of the continuous report tool was 16°.

In Experiment 1, pairs of observers were assigned to control for differences in object states both within and between objects. In each pair, Observer 1 saw Object A in State X in the consistent scene and Object A in State Y in the inconsistent scene, while Observer 2 saw Object A in State Y in the consistent scene and Object A in State X in the inconsistent scene. To control for object repetition, we ensured that objects were not repeated within the first and second halves of the trials. Experiment 1 consisted of 174 trials.

In Experiment 2, each object was repeated three times. To control for target repetition, each object was presented only once per block. Objects were shown in random order and in random states. Experiment 2 consisted of 261 trials in total (three blocks of 87 trials each).

In Experiment 3, each object was presented only once. Based on the results from Experiments 1 and 2, we identified three objects (glasses, medicine, and box) that did not differ in scene consistency ratings between the consistent and inconsistent conditions. These objects were removed from the new experiment. The remaining objects were divided into three groups with similar mean adjustment errors (approximately 3.355). Each group was assigned to a different condition and interleaved across three groups of observers. Each condition included 24 objects. To shorten the experiment and reduce potential interference, we also removed the confidence rating question. Experiment 3 consisted of 84 trials.

### Procedure

Before the experiment, participants completed a virtual chin-rest procedure by adjusting an image of a bank card on the screen to match its physical size^58^. A black fixation cross was presented for 500 ms, followed by a scene alone for 900 ms (200 ms in Experiments 2 and 3), after which an object was presented on top of the scene for an additional 100 ms (see Fig. 1C). A dynamic white noise mask was then shown for 800 ms. Participants reported the state of the object using a continuous report task. We used a wheel adjustment tool similar to those used in orientation and color continuous report paradigms. Each object state was randomly mapped to a specific angle on the wheel, with each state step corresponding to 9.5°. Since state changes are typically linear, the sequence of states was doubled and looped, creating the effect of continuous change (e.g., rotating the wheel clockwise caused a book to gradually open, close, and open again). This approach minimized biases toward reporting extreme states (e.g., fully opened/closed). After the state report, participants rated object-scene consistency (Experiments 1, 2, and 3) and, in Experiments 1 and 2, also reported their confidence using a 1–6 Likert scale. Prior to the main experiment, participants completed a short training session that included a tool demonstration and two practice trials.

###  Data analysis

We computed the error of adjustment as the difference between the reported state and the presented state (with error values ranging from 0 to 19). Importantly, our original preregistered design assumed a repeated-measures ANOVA, the results of which can be found in the Supplementary Materials. We switched to a generalized linear mixed model (GLMM) approach during the review process. To examine the effects of experimental condition on participants’ error, we first inspected the distribution of the dependent variable. As expected, due to the nature of the error calculation, the distribution was right-skewed, with a higher concentration of values near zero. Consequently, conventional linear modeling techniques were inappropriate for this type of distribution. Given that error represents count data, we initially applied a Poisson GLMM with a log link function. The model was specified as follows:

$${\text{error}}\sim {\text{condition}} + \left( {{1}|{\text{participant}}\_{\text{id}}} \right) + \left( {{1}|{\text{object}}\_{\text{name}}} \right) + ({1}|{\text{presented}}\_{\text{state}})$$

In this model, the fixed effect was the experimental condition, and random intercepts were included for participant, object, and presented state to account for variability at these levels.

After fitting the Poisson GLMM to the Experiment 1 data, we checked for overdispersion by calculating the ratio of the sum of the squared Pearson residuals to the residual degrees of freedom. A dispersion ratio greater than 1.5 is indicative of overdispersion. Our analysis yielded a dispersion ratio of 2.7, suggesting that the Poisson assumption of equal mean and variance was violated. In response to this overdispersion, we switched to a negative binomial distribution, which is more flexible for data where the variance exceeds the mean. The negative binomial GLMM was fitted using the same fixed and random effects structure:

$${\text{error}}\sim {\text{condition}} + \left( {{1}|{\text{participant}}\_{\text{id}}} \right) + \left( {{1}|{\text{object}}\_{\text{name}}} \right) + ({1}|{\text{presented}}\_{\text{state}})$$

Comparative assessments using Akaike’s Information Criterion (AIC Poisson—17,787.88; AIC Negative binomial model—15,387.99) and Bayesian Information Criterion (BIC Poisson—17,818.65; BIC Negative binomial model—15,424.92) confirmed that the negative binomial model provided a superior fit relative to the Poisson model. Thus, for all analyses, we used negative binomial GLMM.

To verify the necessity of including all three random effects, we compared the full negative binomial model to a simpler version that only included the random effect for participant_id. The increased AIC and BIC values for the simpler model indicated that the additional random effects (object_name and presented_state) improved model fit (AIC full model—15,387.99; AIC simple model—15,741.57; BIC full model—15,424.92; BIC simple model—15,766.19).

LMM and GLMM statistical analyses were conducted using R^59^. Generalized linear mixed models were fitted using the lme4 package^60^ and lmerTest package^61^. R-squared values were computed using the performance package^62,63^. Data visualization was performed using Seaborn^64^ and Matplotlib^65^.

## Electronic supplementary material

Below is the link to the electronic supplementary material.

Supplementary Material 1

## Data Availability

All data and scene stimuli are publicly available at OSF https://osf.io/kb8my/.
