# Supplementary material for: Scene consistency enhances state representations of real-world objects
Source: Sci Rep. 2025 May 27;15:18581. doi: 10.1038/s41598-025-01662-3 (PMC12116985; doi:10.1038/s41598-025-01662-3)
Supplement: Supplementary file 1 — Supplementary Material 1 [file 41598_2025_1662_MOESM1_ESM.pdf]

Supplementary Materials

Here, we report full results for all negative binomial Generalized linear mixed effects models reported in the manuscript.

Table S1. Experiment 1

error ~ condition + (1|participant_id) + (1|object_name) + (1|presented_state)

	mean error		
Predictors	*Estimate*	*SE*	*p*
(Intercept)	1.00	0.07	**<0.001**
condition [inconsistent]	0.05	0.03	0.106
**Random Effects**			
σ^2	0.65		
$\tau_{00\ \text{object}}$	0.14		
$\tau_{00\ \text{presented state}}$	0.01		
$\tau_{00\ \text{participant\_id}}$	0.04		
ICC	0.23		
Observations	3480		
Marginal R^2 / Conditional R^2	0.001 / 0.226		
AIC	15387.990		

error ~ consistency_report + (1|participant_id) + (1|object_name) + (1|presented_state)

	mean error		
Predictors	*Estimate*	*SE*	*p*
(Intercept)	1.12	0.08	**<0.001**
consistency report	-0.03	0.01	**0.013**
**Random Effects**			
σ^2	0.65		
$\tau_{00\ \text{object}}$	0.14		
$\tau_{00\ \text{presented state}}$	0.01		
$\tau_{00\ \text{participant\_id}}$	0.04		

ICC	0.23
Observations	3480
Marginal R^2 / Conditional R^2	0.002 / 0.227
AIC	15384.484

Table S2. Experiment 2

error ~ condition + (1|participant_id) + (1|object_name) + (1|presented_state)

	**mean error**		
Predictors	*Estimate*	*SE*	*p*
(Intercept)	1.10	0.05	**<0.001**
condition [consistent]	0.03	0.02	0.179
condition [inconsistent]	0.05	0.02	**0.026**
**Random Effects**			
σ^2	0.63		
τ_{00} object	0.10		
τ_{00} participant_id	0.02		
τ_{00} presented state	0.01		
ICC	0.18		
Observations	11484		
Marginal R^2 / Conditional R^2	0.001 / 0.181		
AIC	51835.806		

error ~ consistency_report + (1|participant_id) + (1|object_name) + (1|presented_state)

	**mean error**		
Predictors	*Estimate*	*SE*	*p*
(Intercept)	1.19	0.05	**<0.001**
consistency report	-0.02	0.01	**<0.001**
**Random Effects**			
σ^2	0.63		
τ_{00} object	0.10		

τ_{00} participant_id	0.02
τ_{00} presented state	0.01
ICC	0.18
Observations	11484
Marginal R^2 / Conditional R^2	0.002 / 0.182
AIC	51826.069

Table S3. Experiment 2: Block 1

error ~ condition + (1|participant_id) + (1|object_name) + (1|presented_state)

	mean error		
Predictors	*Estimate*	*SE*	*p*
(Intercept)	1.17	0.06	**<0.001**
condition [consistent]	-0.01	0.04	0.847
condition [inconsistent]	0.08	0.04	**0.029**
**Random Effects**			
σ^2	0.60		
τ_{00} object	0.09		
τ_{00} participant_id	0.02		
τ_{00} presented state	0.02		
ICC	0.17		
Observations	3828		
Marginal R^2 / Conditional R^2	0.002 / 0.174		
AIC	17841.119		

error ~ consistency_report + (1|participant_id) + (1|object_name) + (1|presented_state)

	mean error		
Predictors	*Estimate*	*SE*	*p*
(Intercept)	1.31	0.06	**<0.001**
consistency report	-0.03	0.01	**0.001**

**Random Effects**	
σ^2	0.60
τ_{00} object	0.09
τ_{00} participant_id	0.02
τ_{00} presented state	0.02
ICC	0.17
Observations	3828
Marginal R^2 / Conditional R^2	0.004 / 0.175
AIC	17834.907

Table S4. Experiment 3

error ~ condition + (1|participant_id) + (1|object_name) + (1|presented_state)

	**mean error**		
Predictors	*Estimate*	*SE*	*p*
(Intercept)	1.20	0.05	**<0.001**
condition [consistent]	0.00	0.03	0.921
condition [inconsistent]	0.08	0.03	**0.006**
**Random Effects**			
σ^2	0.60		
τ_{00} object	0.09		
τ_{00} participant_id	0.04		
τ_{00} presented state	0.01		
ICC	0.20		
Observations	6048		
Marginal R^2 / Conditional R^2	0.002 / 0.201		
AIC	28475.422		

error ~ consistency_report + (1|participant_id) + (1|object_name) + (1|presented_state)

	**mean error**		
Predictors	*Estimate*	*SE*	*p*

(Intercept)	1.38	0.06	**<0.001**
consistency report	-0.04	0.01	**<0.001**
**Random Effects**			
σ^2	0.60		
$\tau_{00\ \text{object}}$	0.09		
$\tau_{00\ \text{participant\_id}}$	0.04		
$\tau_{00\ \text{presented state}}$	0.01		
ICC	0.19		
Observations	6048		
Marginal R^2 / Conditional R^2	0.006 / 0.197		
AIC	28455.101		

Table S5. Experiments 2 and 3 combined analysis

error ~ condition + (1|participant_id) + (1|object_name) + (1|presented_state)

	**mean error**		
Predictors	*Estimate*	*SE*	*p*
(Intercept)	1.19	0.05	**<0.001**
condition [consistent]	-0.00	0.02	0.940
condition [inconsistent]	0.09	0.02	**<0.001**
**Random Effects**			
σ^2	0.60		
$\tau_{00\ \text{participant\_id}}$	0.03		
$\tau_{00\ \text{object}}$	0.09		
$\tau_{00\ \text{presented state}}$	0.02		
ICC	0.19		
Observations	9744		
Marginal R^2 / Conditional R^2	0.002 / 0.194		
AIC	45576.951		

error ~ consistency_report + (1|participant_id) + (1|object_name) + (1|presented_state)

	mean error		
Predictors	*Estimate*	*SE*	*p*
(Intercept)	1.35	0.05	**<0.001**
consistency report	-0.04	0.01	**<0.001**
**Random Effects**			
σ^2	0.60		
$\tau_{00\ participant\_id}$	0.03		
$\tau_{00\ object}$	0.09		
$\tau_{00\ presented\ state}$	0.02		
ICC	0.19		
Observations	9744		
Marginal R^2 / Conditional R^2	0.006 / 0.192		
AIC	45553.747		

In the following section, we report original RM ANOVA and regression analysis, as it was preregistered and was performed before review.

Experiment 1

Mean error

We analyzed the mean error of adjustment as a difference in “frames” between the reported state and the originally presented state. We found a significant difference in the mean error of adjustment between consistent and inconsistent conditions (Fig. 2A, $t(19) = 2.168$, $p = 0.043$, Cohen's d = 0.25). Additionally, we found a significant effect of individual observers' consistency reports on the mean error (Fig. 2B, linear regression, error ~ consistency report, $p = 0.04$, $R^2 = 0.001$), with the error decreasing with an increase in the reported consistency. Unsurprisingly, the error was also smaller for confident reports (linear regression, error ~ confidence report, $p < 0.001$, $R^2 = 0.056$).

Consistency and confidence reports

Participants reported that consistent scenes are more consistent compared to inconsistent ones ($t(19) = 6.6$, $p < 0.001$, Cohen's d = 2.13). Also, confidence reports were slightly higher for consistent compared to inconsistent condition ($t(19) = 2.8$, $p = 0.011$, Cohen's d = 0.225).

Experiment 2

Mean Error

We found a significant but unreliable effect of the condition on the mean error of adjustment (Fig. 3A, RM ANOVA, $F(2, 86) = 3.129$, $p = 0.048$, $\eta^2_p = 0.068$) with numerically increased errors in the inconsistent condition. We also replicated a significant effect of individual observers' consistency reports on the mean error (Fig. 3B, linear regression, error ~ consistency report, $p = 0.001$, $R^2 = 0.0008$).

Consistency and confidence reports

As expected, consistency reports were affected by object-scene consistency (RM ANOVA, $F(2, 86) = 146.6$, $p < 0.001$, $\eta^2_p = 0.77$) in that participants reported that consistent scenes were more consistent compared to inconsistent ($t(43) = 12.38$, $p_{holm} < 0.001$) and control ($t(43) = 12.58$, $p_{holm} < 0.001$), while inconsistent trials had the lowest consistency ratings ($t(43) = 10.82$, $p_{holm} < 0.001$). Additionally, we found a significant effect of object-scene consistency on confidence reports (Fig. 3C, RM ANOVA, $F(2, 86) = 5.84$, $p = 0.004$, $\eta^2_p = 0.12$), with increased reported confidence for consistent trials (consistent vs. inconsistent comparison: $t(43) = 3.2$, $p_{holm} = 0.007$).

Our analysis revealed that block number or target repetition significantly affected the mean error (RM ANOVA, condition – $F(2, 86) = 2.870$, $p = 0.062$, $\eta^2_p = 0.06$; block number – $F(2, 86) = 9.502$, $p < 0.001$, $\eta^2_p = 0.18$; interaction - $F(4, 174) = 0.759$, $p = 0.554$, $\eta^2_p = 0.02$), with block number one having the largest error (block 1 vs block 2: $t(43) = 3.53$, $p_{holm} = 0.003$; block 1 vs block 3: $t(43) = 3.41$, $p_{holm} = 0.003$).

Experiment 3

Mean error

We found a significant effect of consistency on the mean error of adjustment (Fig. 5A, RM ANOVA, $F(2, 142) = 3.369$, $p = 0.027$, $\eta^2_p = 0.049$) with only numerically increased errors in the inconsistent condition. We also replicated a significant effect of individual observers' consistency reports on the mean error (Fig. 5B, linear regression, error ~ consistency report, $p < 0.001$, $R^2 = 0.1$).

Consistency reports

Again as expected, consistency reports were affected by consistency condition (RM ANOVA, $F(2, 142) = 57.98$, $p < 0.001$, $\eta^2_p = 0.45$) in that consistent scenes were reported as more consistent compared to inconsistent ($t(71) = 8.15$, $p_{holm} < 0.001$) and control ($t(71) = 7.17$, $p_{holm} < 0.001$), while inconsistent trials had the lowest consistency ratings ($t(71) = 6.47$, $p_{holm} < 0.001$).

Experiments 2 and 3 combined analysis

To increase the power of our analysis, we combined data from Experiments 2 and 3, including only the first block of Experiment 2 and excluding categories not presented in Experiment 3 ("*glasses*," "*medicine*," and "*box*"). This resulted in a sample size of 116 participants and substantial effect of consistency on the mean error of adjustment (Fig. 6A, RM ANOVA, $F(2, 230) = 6.006$, $p = 0.003$, $\eta^2_p = 0.049$) with significantly higher errors of adjustment for inconsistent objects (consistent vs. inconsistent: $t(115) = 2.82$, $p_{holm} = 0.01$; control vs inconsistent: $t(115) = 2.96$, $p_{holm} = 0.01$; consistent vs control: $t(115) = 0.05$, $p_{holm} = 0.959$). We also found a significant effect of individual observers' consistency reports on the mean error (Fig. 6B, linear regression, error ~ consistency report, $p < 0.001$, $R^2 = 0.006$).
